# Executive Functions in Children Who Experience Bullying Situations

**DOI:** 10.3389/fpsyg.2016.01197

**Published:** 2016-08-26

**Authors:** Wandersonia Medeiros, Nelson Torro-Alves, Leandro F. Malloy-Diniz, Carla M. Minervino

**Affiliations:** ^1^Laboratory of Cognitive Sciences and Perception-Laboratory of Mental Health, Education and Psychometric, Universidade Federal da ParaíbaParaíba, Brazil; ^2^Postgraduate Program in Cognitive Neuroscience and Behaviour, Laboratory of Cognitive Sciences and Perception, Universidade Federal da ParaíbaParaíba, Brazil; ^3^ILUMINA Neurosciences, LINC-INCT, Universidade Federal de Minas GeraisBelo Horizonte, Brazil; ^4^Postgraduate Program in Cognitive Neuroscience and Behaviour, Laboratory of Mental Health, Education and Psychometric, Universidade Federal da ParaíbaParaíba, Brazil

**Keywords:** bullying, decision-making, executive function, aggressive behavior, cognitive flexibility

## Abstract

Bullying is characterized by intentional, repetitive, and persistent aggressive behavior that causes damage to the victim. Many studies investigate the social and emotional aspects related to bullying, but few assess the cognitive aspects it involves. Studies with aggressive individuals indicate impairment in executive functioning and decision-making. The objective of this study was to assess hot and cold executive functions in children who experience bullying. A total of 60 children between 10 and 11 years of age were included in the study. They were divided into four groups: aggressors (bullies), victims, bully-victims, and control. Tests for decision-making, inhibitory control, working memory, and cognitive flexibility were used. The bully group made more unfavorable choices on the Iowa Gambling Task, which may indicate difficulties in the decision-making process. The victim group took longer to complete the Trail Making Test (Part B) than aggressors, suggesting lower cognitive flexibility in victims. The hypothesis that aggressors would have lower performance in other executive functions such as inhibitory control, working memory, and cognitive flexibility has not been confirmed. This study indicates that bullies have an impairment of hot executive functions whereas victims have a comparatively lower performance in cold executive functions. In addition to social and cultural variables, neurocognitive and emotional factors seem to influence the behavior of children in bullying situations.

## Introduction

The word bullying is used to characterize intentional repetitive and persistent aggressive behavior toward a victim (Olweus, 1994). There is an uneven power relationship between the aggressor and the victim in bullying due to differences in age, physique, or strength. This difference sustains the behavior of the bully even despite clear signs of discomfort and displeasure on the part of those suffering from it (Smith, 2002).

Bullying aggression can occur by direct physical contact (kicking, punching, pushing, theft, or damage to the victim’s objects), psychological aggression (verbal abuse involving nicknames, insults, or mean comments about race, sexuality, religion, and physical features) or indirectly (excluding the victim from playing or group conversations) (Bullock, 2002).

In a bullying situation, children can take on different roles. Aggressing children (bullies) have the intention of causing harm or excluding others (Berger, 2007). Children that are victims suffer from the constant aggression and often fail to react or get others to stop. Children considered victims-aggressors are those that bully/offend but also suffer aggression, and they differ from aggressors because they are not as popular and usually replicate the aggression with a more fragile child (Bandeira and Hutz, 2012). Bystanders are those children who are not directly involved in the aggression but those who witness bullying. Bystander often do not know how to behave in the face of aggression and become silent for fear of becoming victims, or for not trusting the actions taken by school professionals (Lopes Neto, 2005; Miranda, 2011).

The prevalence of bullying seems to vary depending on the sociocultural context. For example, Berger (2007) reviewed the prevalence of bullying in different countries and found a range between 3 and 27% of bullies and between 9 and 32% of victims. However, methodological differences and the definition of bullying itself make it difficult to establish epidemiological comparisons. Understanding the psychological processes involved in the onset and maintenance of a bullying relationship involves clarifying the cognitive and personality factors of bullies and victims. Koh and Wong (2015) argue that the psychological traits of the aggressors reflect potential adaptive advantages related to sexual selection. However, there is evidence that cognitive deficits and certain personality traits are most frequently found in children and adolescents who bully (Medeiros et al., 2014).

Children who are bullies and bully-victims show more frequent antisocial behavior and lower levels of empathy compared to victims and children who do not experience bullying (Camodeca and Goossens, 2005; Gery et al., 2009; Viding et al., 2011). They also have lower academic performance, an increased school drop-out rate, and higher involvement with the justice system (Gini, 2006). Coolidge et al. (2004) observe that bullying behavior is associated with deficits in executive functioning, conduct disorders, oppositional-defiant disorder, attention deficit hyperactivity disorders (ADHDs), and increased use of substances such as alcohol and marijuana.

Regarding executive functions, Diamond (2013) believes that these functions involve three main centers: inhibitory control, working memory, and cognitive flexibility. According to the author, the other functions such as reasoning, planning and organization are built from these three functions.

Currently, some authors have distinguished executive functions into cold and hot. Cool executive functions are related to cognitive/rational high-order process and are used to general cognitive control. Hot executive functions, in turn, are cognitive/emotional processes related to affective decision making, motivation, and social cognition. According to Damásio (1996), decision-making processes are related to the interpretation of body states and emotional bias defined as somatic markers. The process of somatic markers interpretation is important both to risk perception and decision considering immediate and future outcomes. Antisocial behavior has been associated to impairment in somatic marker processing (Damásio et al., 1996; Séguin, 2004; Sinclair and Gansler, 2006; Tung and Chhabra, 2011). Despites the knowledge of social rules, antisocial subjects present rule-breaking behaviors due to the lack of interpretation of these emotional-somatic signals.

Verlinden et al. (2014) found that children who are not involved in bullying situations as bullies or victims have better scores on intelligence tests. Bullies, victims, and bully-victims have greater difficulty with inhibitory control according to the reports of their parents in the BRIEF Scale. This result suggests a probable deficit in executive functioning related to involvement in bullying situations. Such results, however, have certain limitations. Verlinden et al. (2014) use indirect measures of executive functioning in questionnaires that assess parents’ perceptions of such cognitive processes.

Cognitive-emotional aspects of executive functions are poorly investigated in studies on bullying, however, they are particularly important in regulating behavior in social situations (Smith and Jones, 2012). Affective decision-making seems to be related to the presence of various psychopathological conditions such as ADHD; autism spectrum disorders, substance abuse such as alcohol and/or cigarettes; conduct disorders; schizophrenia, as well as behavioral problems such as high disinhibition, self-harm, and aggressive behavior (Best et al., 2002; Ernst et al., 2003, 2010; Verdejo-García et al., 2006; Suhr and Tsanadis, 2007; DeVito et al., 2008; Fairchild et al., 2009; Herrera, 2011; Mata et al., 2011; Sallum et al., 2013). Bullies often have alterations in behavior, therefore, the possibility that decision-making may also be impaired in these individuals cannot be ruled out.

In view of the likely involvement of executive functioning in different behavioral patterns related to bullying, the objective of this study was to evaluate the different components of these functions in groups of bullies, victims, bully-victims, and a control group.

As a hypothesis, we consider that bullies, victims and bully-victims child groups would achieve lower scores on the evaluation of executive functions than the group that has no direct involvement with bullying. Another hypothesis was that the group of aggressors and victims-aggressors would demonstrate lower performance on inhibitory control and decision-making in comparison to victims and controls.

## Materials and Methods

### Sample

Initially, the Peer Aggression and Victimization Scale (PAVS) was applied to 225 students of the 6th grade of two public schools and a private school. After the exclusion of children who did not fit in the pre-established age group, the scales and the free informed consent form were delivered to parents. Children were recruited according to the results of the PAVS scale. Children were excluded if they were 12 years or older (as they would be at a different phase of development, in this case in adolescence), with complaints of uncorrected visual or hearing difficulties and/or those with compromising cognitive impairment. Thirty-nine children completed the individual steps, however, they were not included in data analysis because their parents did not deliver the Strengths and Difficulties Questionnaire.

The sample comprised 60 children (32 females and 28 males), with age between 10 to 11 years and attending sixth grade in middle school. A total of 34 children attended private schools and 26 attended public schools in João Pessoa.

### Instruments

#### Strengths and Difficulties Questionnaire – SDQ (Goodman, 1997)

The instrument used to characterize the sample was the Strengths and Difficulties Questionnaire- SDQ, developed by Goodman (1997) and validated for the Brazilian context by Fleitlich et al. (2000). It is a screening questionnaire that aims to assess mental health of children and adolescents (4–16 years). It has 25 items, divided into five subscales: emotional symptoms, conduct problems, hyperactivity, relationship problems with peers and prosocial behavior. A total index of difficulty which is the sum of the subscales (except sociability) is also generated. The instrument can be used in three versions (self-reporting, a version for parents and a version for teachers). In the present study, we used the version for parents. Each item has three alternatives, false (zero), more or less true (one point) and true (two points).

#### Bullying Evaluation: Peer Aggression and Victimization Scale (PAVS; Cunha et al., 2009)

The PAVS is a self-reported, 18-item scale, applicable to students attending the second half of elementary school (in general, children from 10 to 15 years of age), that investigates behaviors of bullying and victimization among peers. The child selects the frequency (never, rarely, sometimes, very often, or always) with which they performed or suffered a certain behavior at school during the previous 6 months. The answers were added separately according to the following classification: direct aggression, relational aggression, indirect physical aggression, and victimization. This study did not investigate “indirect physical aggression.” From the sum of the scores in each class, participants were divided into subgroups according to the cutoff points (Cunha et al., 2009). The scale was used to classify participants into groups of bullies, victims, and bully-victims, in addition to a control group, by using the following criteria:

(a) Bully group – This group comprises children with a high frequency of behavior in the direct aggression items (score ≥ 9) and low (score ≤ 12) or moderate (score ≤ 16) behavior in the victimization items. Children with high scores only in the relational aggression items were not included.
(b) Victim group – This group comprises children with a high frequency of victimization behavior (score ≥ 16) and a low level of direct physical aggression (score ≤ 7) and relational aggression (score ≤ 6).
(c) Bully-victim group – This group comprises children with a high frequency of direct aggressive behavior (score ≥ 9) and victimization (score ≥ 16). Children with a high score in victimization and high scores only in relational aggression were not included.
(d) Control group – This group comprises children with a low frequency of direct aggressive behavior (score ≤ 7) and victimization with a low (score ≤ 12) or moderate (score < 16) frequency.

### Evaluation of Executive Functioning

The protocol for the analysis of cold executive functions used the model proposed by Diamond (2013). In this model, three main cores are part of executive functioning: inhibitory control, working memory, and cognitive flexibility. These three functions give rise to the other functions, such as reasoning, planning, and organization. The following instruments were used.

#### Digit Span Backward (DSB) Subtest (Wechsler, 2013)

A subtest of the Wechsler Intelligence Scale for Children (WISC-IV), the DSB evaluates working memory. For this task, the professional applying the test reads some numbers aloud, and the child must repeat them in descending order. The tables for 10 and 11 years of age were used to transform the raw scores into weighted scores.

#### Trail Making Test Part B (TMT-B)

The TMT has two parts: A and B. This study used only part B, which evaluates alternating attention and mental flexibility. The child was instructed to connect the numbers in ascending order and in alphabetical order. The execution time of the activity was considered to determine the score. When there was an error, the evaluator showed it to the participant and requested correction, which increased the execution time.

#### Victoria Stroop Color-Word Test

The Stroop Test was used to assess attention and inhibitory control. The Victoria version includes three cards, 1 (color), 2 (word), and 3 (color-word). The first card (color) contains colored rectangles with the colors pink, green, blue, and brown, which must be named by the child as quickly as possible. The second card (word) lists the words EACH, TODAY, NEVER, and EVERYTHING, printed in the same colors, and the child is asked to simply read the words as quickly as possible. The third card (color-word) lists the names of the four colors, printed in colors that are incompatible with the written word (e.g., the word “Blue” is printed in pink). The child is asked not to read the names but instead to name the printed colors as quickly as possible. For the assessment, the execution time for each card and the number of mistakes (errors not spontaneously corrected by the child) were taken into account (Kulaif, 2005).

For the assessment of the “hot” executive functions, the test of affective decision-making described below was used.

#### Iowa Gambling Task (IGT; Bechara et al., 1994)

The Brazilian version of the IGT, adapted by Malloy-Diniz et al. (2008), was used to evaluate hot executive functions as a test of emotional decision-making. In this task, participants aim to achieve the maximum gain from an initial cash loan. Individuals make 100 selections of cards, not knowing in advance how many are allowed, and they must make decisions that lead to a final positive result according to the feedback that they receive. Card decks A and B initially offer an advantage, but in the long term, they become unfavorable. Decks C and D offer an overall advantage because, although they have a lower value of rewards, the punishments are also smaller, ultimately resulting in a higher overall gain.

In this study, to increase the chances that the test evaluated hot executive functions, a reward (candy) was included, according to the following criteria:

• Gains of 25, 50, and 75 (frequent in advantageous decks): receives one piece of candy;
• Losses of 25, 50, and 75 (frequent in advantageous decks): loses one piece of candy;
• Gains ≥ 100 (frequent in disadvantageous decks): receives two pieces of candy;
• Losses ≥ 100 (frequent in disadvantageous decks): loses two pieces of candy; and
• Losses = 1500 (only in disadvantageous decks): loses all candy.

### Procedures

After approval by the school and parents by means of agreement documents and free and informed consent forms, the groups were determined by applying the PAVS. For the individual step, tests for the assessment of executive functioning were applied in an isolated room arranged by the school. The professional applying the test was with the child throughout task execution to answer questions and prevent mistakes due to confusion.

The study was approved by the Research Ethics Committee of the University Hospital Lauro Wanderley (Hospital Universitário Lauro Wanderley), UFPB (CAAE process: 17883413.5.0000.5183). All procedures were performed according to Regulation 466/96 of the National Health Council (Conselho Nacional de Saúde – CNS). Participation was voluntary, and the participants were informed in advance that they could withdraw their consent at any time during the study.

### Statistical Analysis

The softwares SPSS 21.0 and Microsoft Office Excel 2007 were used for the tabulation of data and statistical analysis. The Shapiro–Wilk test and Levene’s test showed that data did not present normal distribution and equality of variances (homoscedasticity), respectively. For this reason, non-parametric testing was selected. The Kruskal–Wallis test was used for comparisons between groups using a significance level of 0.05. When Kruskal–Wallis test was inferior to 0.05, we performed Mann–Whitney pairwise comparisons. In order to control the probability of Type I error, we corrected the critical value of alfa by dividing the familywise error rate (0.05) by the number of comparisons (6). Therefore, we considered as statistically significant only the probabilities values inferior to 0.0083.

In addition, we determined effect sizes estimates (Fritz et al., 2012; Field, 2013). As proposed by Cohen (1988), effect sizes were calculated using the formula:

$$r=\frac{z}{\sqrt{N}}\,\;\;\;\;\;\;\;\;\;\;\;\;\;\;\;\;\;\;\;\;\;\;\;\;\;\;\;\;\;\;\;\left(1\right)$$

where *r* is the effect size estimate, *z* is the standard score of the distribution, and *N* is the total of the sample size.

According to Cohen’s guidelines used to interpret *r*, a large effect is superior to 0.5, a medium effect is 0.3 and a small effect is 0.1. Large effect sizes associated to non-significant results may suggest to carry out a research with a greater power, whereas small effect sizes associated to significant results may indicate that the observed effects are not so robust (Fritz et al., 2012).

## Results

With regard to socio demographic characteristics, a total of 32 out of the 60 children who participated in the sample were female, and 28 were male, with 34 attending private schools and 26 attending public schools in João Pessoa, Paraíba, Brazil.

The participants were divided into four groups according to their result on the PAVS scale: bullies (*n* = 15; seven male), victims (*n* = 15; six male), bully-victims (*n* = 15; nine male), and control (*n* = 15; six male). A preliminary statistical analysis showed no differences between genders with regard to the variables investigated in the study (*p* > 0.05). **Table 1** shows the average score of the four groups in the dimensions of the PAVS scale. In **Table 2**, we present data of the four groups of participants and results of the statistical analysis.

**Table 1:** Means and standard deviations of age and scores in the PAVS scale dimensions in the four groups of participants.

Variable	Bully	Victim	Bully-Victim	Control
Age	10.73 (0.45)	10.87 (0.35)	10.80 (0.41)	10,73 (0.45)
Relacional aggression	8.20 (4.29)	5.93 (1.62)	8.53 (3.29)	4.87 (1.12)
Direct aggression	11.73 (2.01)	6.47 (0.91)	12.67 (1.71)	6.40 (1.18)
Victimization	13.33 (2.19)	20.73 (4.90)	23.73 (5.78)	11.07 (2.05)

**Table 2:** Statistical analysis and scores obtained by the four groups of participants in the Strengths and Difficulties Questionnaire (SDQ), Digit Span Backward Subtest (DSB), Trail Making Test part B (TMT-B), Victoria Stroop Color-Word Test (STROOP), Iowa Gambling Task (IGT).

Instrument	Bully	Victim	Bully-Victim	Control	Kruskal–Wallis
**SDQ**					
Emotional symptoms	3.53 (2.26)	5.80 (3.07)	3.27 (2.86)	4.00 (2.77)	*p* = 0.086
Conduct problems	3.07 (2.65)	1.87 (1.80)	2.73 (1.43)	2.27 (1.62)	*p* = 0.472
Hyperactivity	4.60 (3.26)	3.53 (2.10)	4.33 (1.91)	3.93 (2.89)	*p* = 0.780
Peer relationship Problems	1.53 (1.72)	2.67 (1.63)	2.47 (2.56)	1.20 (1.01)	*p* = 0.074
Prosocial behavior	7.07 (2.40)	9.27 (0.96)	7.87 (1.59)	8.33 (1.54)	*p* = 0.010ˆ*
Total difficulties	12.80 (8.24)	14.07 (7.16)	12.80 (6.71)	11.40 (5.27)	*p* = 0.780
**DSB**	9.33 (2.52)	9.53 (2.35)	9.93 (1.94)	8.73 (2.12)	*p* = 0.460
**TMT-B**	40.27 (10.27)	61.40 (17.31)	48.60 (12.43)	48.20 (11.91)	*p* = 0.003ˆ*
**STROOP**					
Color (Time)	16.00 (0.92)	19.13 (0.89)	18.58 (1.21)	17.80 (1.03)	*p* = 0.126
Color (Errors)	0.00 (0.00)	0.07 (0.26)	0.00 (0.00)	0.13 (0.352)	*p* = 0.284
Word (Time)	11.27 (3.41)	12.40 (2.41)	12.26 (2.68)	12.67 (2.58)	*p* = 0.205
Word (Errors)	0.07 (0.26)	0.00 (0.00)	0.13 (0.51)	0.07 (0.26)	*p* = 0.792
Color-word (Time)	27.47 (1.68)	34.20 (3.25)	35.21 (2.76)	30.40 (1.84)	*p* = 0.018ˆ*
Color-word (Errors)	0.40 (0.63)	0.27 (0.59)	0.47 (1.12)	0.60 (1.24)	*p* = 0.869
**IGT**					
Deck A choices	25.15 (0.74)	22.60 (1.29)	20.79 (0.96)	21.07 (1.26)	*p* = 0.039ˆ*
Deck B choices	27.33 (1.55)	27.53 (1.29)	30.13 (1.60)	29.47 (1.57)	*p* = 0.286
Deck C choices	25.00 (1.17)	25.00 (0.99)	26.47 (1.04)	24.53 (0.66)	*p* = 0.325
Deck D choices	24.00 (1.82)	24.87 (1.94)	21.73 (1.37)	24.93 (1.57)	*p* = 0.566
General Trend Block 1 (1–20)	-0.93 (0.64)	-1.47 (0.74)	-1.47 (0.90)	-1.07 (0.54)	*p* = 0.971
General trend block 2 (21–40)	-1.20 (0.95)	-0.40 (1.58)	-3.07 (1.53)	-1.47 (0.71)	*p* = 0.859
General trend block 3 (41–60)	-0.27 (0.67)	1.47 (1.75)	-2.00 (1.08)	1.07 (1.40)	*p* = 0.697
General trend block 4 (61–80)	-0.13 (1.50)	-0.93 (1.24)	0.80 (1.59)	-0.67 (1.11)	*p* = 0.640
General trend block 5 (81–100)	0.53 (0.95)	1.07 (0.93)	2.13 (1.38)	1.07 (1.18)	*p* = 0.995
General trend	-2.00 (3.20)	-0.27 (3.95)	-3.60 (3.48)	-1.07 (3.03)	*p* = 0.881

*Values correspond to means and standard deviations.*

### Strengths and Difficulties Questionnaire – SDQ

We found no differences between groups with regard to the total score of difficulties (χ^2^ = 1.088, *p* = 0.780), emotional problems (χ^2^ = 6.585, *p* = 0.086), hyperactivity (χ^2^ = 1.087, *p* = 0.780), conduct problems (χ^2^ = 2.517, *p* = 0.472) and peer relationship problems (χ^2^ = 6.920, *p* = 0.740). In the prosocial behavior subscale, we found differences between groups (χ^2^ = 11.347, *p* = 0.01). Mann–Whitney tests showed that victims presented higher scores than bullies (*U* = 33.00, *z* = -3.217, *p* = 0.001, *r* = -0.59), but not in comparison to the control group (*U* = 73.5, *z* = -1.703; *p* = 0.089, *r* = -0.31) and bully-victims (*U* = 54.5, *z* = -2.495; *p* = 0.013, *r* = -0.46). Bullies, bully-victims and controls had similar scores in prosocial behavior compared one another.

### Digit Span Backward (DSB) Subtest

There was no significant difference between the groups with regard to DSB (χ^2^ = 2.587, *p* = 0.46), indicating that they had similar patterns of performance in working memory.

### Trail Making Test Part B (TMT-B)

**FIGURE 1:**
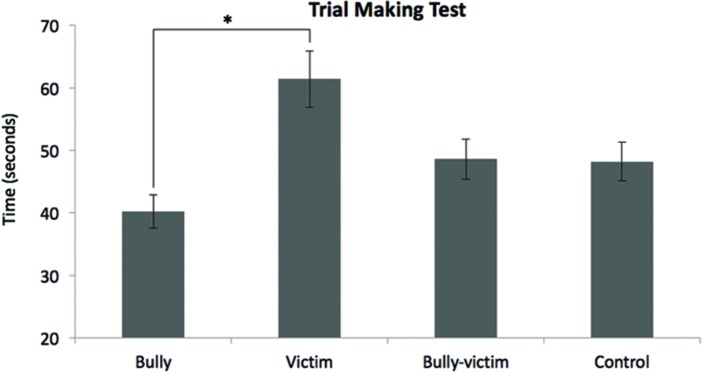
**Time, in seconds, on the Trail Making Test Part B (TMT-B).** ^∗^Significant difference for the victim group compared to the bully group (*p* = 0.001).

Statistical analysis showed significant differences between groups in TMT-B (χ^2^ = 13.839, *p* = 0.003). Mann–Whitney tests showed that the victim group had a longer execution time in TMT-B compared to bullies (*U* = 32.5, *z* = -3.322; *p* = 0.001, *r* = -0.61), but not differed from bully-victims (*U* = 65, *z* = -1.972, *p* = 0.049, *r* = -0.36), and controls (*U* = 62.5, *z* = -2.075, *p* = 0.038, *r* = -0.38). This result indicates a lower cognitive flexibility in the victim group. The other groups did not differ one another: aggressors compared to bully-victims (*U* = 66, *z* = -1.932, *p* = 0.053, *r* = -0.35); bullies compared to controls (*U* = 66, *z* = -1.93, *p* = 0.054, *r* = -0.35); bully-victims compared to controls (*U* = 109.5, *z* = -0.125, *p* = 0.901, *r* = -0.02; **Figure 1**).

### Stroop Color-Word Test

**FIGURE 2:**
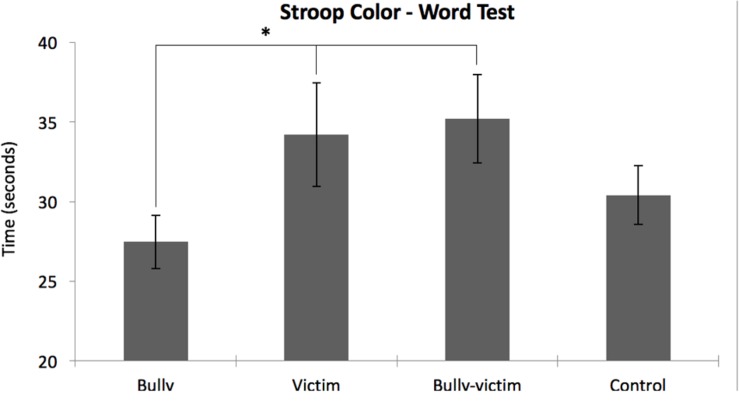
**Time, in seconds, on the Stroop Color-Word Test.** ^∗^Significant difference for the bully group compared to victim (*p* = 0.006) and bully-victim (*p* = 0.006).

The Kruskal–Wallis test showed significant differences between groups only for the third card (color-word; χ^2^ = 10,039, *p* = 0.018). Mann–Whitney tests showed that the bully group had a shorter execution time compared to the victim (*U* = 35, *z* = -2.736, *p* = 0.006, *r* = -0.50) and bully-victim groups (*U* = 31, *z* = -2.754, *p* = 0.006, *r* = -0.50), but not differed from control group (*U* = 52, *z* = -2.109, *p* = 0.035, *r* = -0.39; **Figure 2**). In the third card, we found no differences between victims compared to bully-victims (*U* = 87, *z* = -0.195, *p* = 0.846, *r* = 0.04); victims compared to controls (*U* = 93, *z* = -0.525, *p* = 0.600, *r* = 0.10); bully-victims compared to controls (*U* = 83, *z* = -0.669, *p* = 0.503, *r* = 0.12). For the first card (color), we found no statistically significant differences between groups (χ^2^ = 5.718; *p* = 0.126), but victims presented longer execution time than other groups. We find no differences between groups with regard to Stroop word (time; χ^2^ = 4.587, *p* = 0.205), Stroop color (errors; χ^2^ = 3.795, *p* = 0.284), Stroop word (errors; χ^2^ = 1.037, *p* = 0.792), and Stroop color-word (errors; χ^2^ = 0.718, *p* = 0.869) (**Figure 2**).

### Iowa Gambling Task – IGT

**FIGURE 3:**
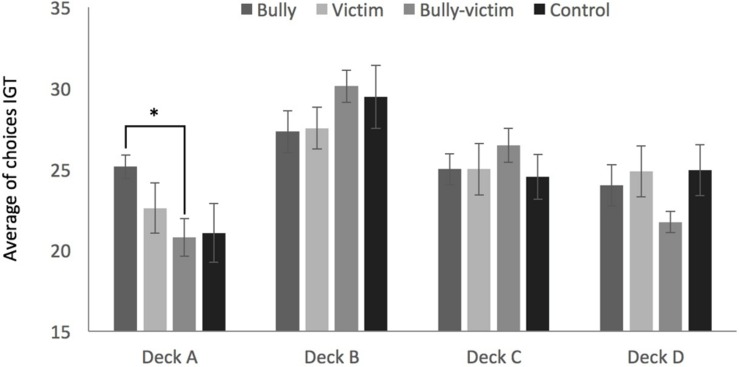
**Average of choices from decks A, B, C, and D on the Iowa Gambling Task (IGT).** ^∗^Significant difference for the bully group compared to the bully-victim group in Deck A (*p* = 0.006).

The Kruskal–Wallis test showed significant differences between groups only for choices from Deck A (χ^2^ = 8,393, *p* = 0.039). Mann–Whitney tests showed that the bully group had the highest score considering all choices from Deck A compared to the bully-victim group (*U* = 34.5 *z* = -2.755, *p* = 0.006, *r* = 0.50), but not differed from victims (*U* = 61.5 *z* = -1.666, *p* = 0.96, *r* = 0.30) and the control group (*U* = 49, *z* = -2.246, *p* = 0.025, *r* = 0.41). Other groups had a similar number of choices of cards in Deck A: victims compared to bully-victims (*U* = 82, *z* = -1.006, *p* = 0.314, *r* = 0.18); victims compared to controls (*U* = 94, *z* = -0.771, *p* = 0.440, *r* = 0.14); bully-victims compared to controls (*U* = 99.50, *z* = -0.241, *p* = 0.810, *r* = 0.04). There was no significant differences between the groups with regard to the general score trends in blocks (χ^2^ = 0.241, *p* = 0.971 in Block 1; χ^2^ = 0.758, *p* = 0.859 in Block 2; χ^2^ = 1.435, *p* = 0.697 in Block 3; χ^2^ = 1.685, *p* = 0.640 in Block 4, and χ^2^ = 0.096, *p* = 0.995 in Block 5) and the overall general trend (χ^2^ = 0.668, *p* = 0.881). There was also no difference between the total number of choices from Decks B (χ^2^ = 3.780, *p* = 0.286), C (χ^2^ = 3.466, *p* = 0.325), and D (χ^2^ = 2.031, *p* = 0.566) (**Figure 3**).

## Discussion

In this study, executive functioning (inhibitory control, working memory, and cognitive flexibility) and emotional decision-making were assessed in children who experience bullying. This study is innovative because it investigates multiple components of executive functioning and their relationship to bullying.

We used the Strengths and Difficulties Questionnaire – SDQ (parent version) to characterize chid behavior. In the SDQ, offending children (bullies) did not differ from other groups with regard to the symptoms of hyperactivity and behavior problems, contradicting the results of Coolidge et al. (2004), which found a higher prevalence of challenging behavior, conduct problems and ADHD in offending children compared to the control group. Therefore, it is clear that aggressors of this sample did not differ from controls with respect to behavior problems, probably due to this group presenting many children who also had average levels of victimization. Viding et al. (2011) claim that not all behavior problems are due to the same cause, so it is important to investigate the profile of each group.

The group of victims obtained the highest score of prosocial behavior, related to behaviors of empathy (helping others, being nice, and caring for younger children) than the group of bullies. However, there were no differences between victims compared to controls and bully-victims.

Deficits in executive functioning have been reported in several developmental and behavioral disorders (Lezak, 1982; Hamdan and Pereira, 2009). In this study, there was no significant difference between groups with regard to working memory, assessed by DSB Subtest. With regard to working memory, Best et al. (2002) found no differences between patients with aggressive and impulsive behavior compared to controls. Nevertheless, they observed that the clinical group showed impairment in the decision-making process.

Contrary to expectations, there was no deficit in inhibitory control functions and cognitive flexibility in bullies, as assessed by the Stroop test and TMT-B, respectively. The bully group had a shorter execution time on the TMT-B compared to the victims group and the third Stroop card (color-word) compared to the victims and bully-victims groups and not presented more errors. Brennan (2002) found no association between high levels of juvenile delinquency and poor performance in the executive function assessment tasks. However, our results are different from those reported by other authors who observed inhibitory control difficulties in aggressive individuals (Best et al., 2002; Ellis et al., 2009). Verlinden et al. (2014) found a relationship between low inhibitory control and involvement in bullying, whether in the bully, victim or bully-victim roles. Nevertheless, the authors used an indirect assessment with questionnaires directed to parents, which may explain the difference between the results. Compared to bullies, the victim group had a longer TMT-B execution time, indicating less efficient performance in the context of cognitive flexibility. Although not statistically significant, we found medium effect sizes to the comparisons between victims and bully-victims (*r* = 0.36) and between victims and controls (*r* = 0.38), what indicates a reduction in cognitive flexibility in victims compared to those groups. This result is consistent with those found by Dertelmann (2011), who found lower performance in cognitive flexibility tests and working memory in child victims of abuse (Dertelmann, 2011). Brennan (2002) found that children who had suffered abuse and higher delinquency levels had lower scores on the assessment of executive functioning. This result shows that the presence of victimization is a factor that may be associated with deficits in the development of cognitive impairment. Other studies also show worse performance in tasks that assess executive functioning in individuals who have suffered physical or sexual assaults (Stein et al., 2002; Coolidge et al., 2004).

The association between deficits in executive functioning and vulnerability to aggression can be explained as a consequence of exposure to violence, but it can also be understood as a factor of vulnerability to such acts. The different types of victimization investigated and the lack of longitudinal studies do not allow us to safely determine whether impairment in executive functioning is a result of aggression, whether it is associated with psychiatric disorders developed due to aggression (anxiety disorders, post-traumatic stress disorder), or whether it is part of the cognitive and personality features of these individuals, which may predispose them to suffer aggression (Stein et al., 2002). According to Teicher et al. (2003), exposure to violence or severe stress in childhood, depending on its severity and intensity, can cause neurobiological changes and affect brain development. For example, Johnson (2012) suggests that the presence of deficits in executive functioning since childhood can make individuals less efficient in problem solving and, therefore, less resilient in adverse situations.

In this study, compared with the bully-victim group, children in the bully group choose more cards from one of the disadvantageous decks (Deck A). Decks A and B are considered disadvantageous because they cause losses over the long term of task execution compared to decks C and D. Although deck B is also unfavorable, Deck A has the highest frequency of punishments but is lower in intensity (Singh, 2013). We found no statistically significant differences between bullies and controls in IGT, however, the effect size obtained in the comparison (0.41) indicates that groups might differ each other in studies involving a greater sample, with bullies choosing more disadvantageous cards.

This finding suggests that bullies are more sensitive to the intensity of punishment than they are to the frequency of punishment. The choice for one of the unfavorable decks by bullies indicates a preference for immediate gains (two pieces of candy), ignoring punishment and the future consequences of the choice. Such behavior has been designated “myopia for the future” (Bechara et al., 1994, 2002; Gomes et al., 2011).

With regard to “myopia for the future,” the following analogy can be made for a situation experienced at school: a child can choose between advantageous attitudes (not attacking), which generate long-term gains (e.g., being aware of doing the right thing, building lasting and true friendships), and unfavorable behavior (aggression), which brings immediate gains (a sense of power, entertaining peers, being popular) but generate losses in the long run (complaints, punishment by parents, poor school performance, and shallow friendships). In this sense, it is possible to consider that the practice of bullying in the school context may be associated with a decrease in the decision-making process of bullies. Decision-making deficits are clearly found in children and adolescents with behavioral disorders, conduct disorders and in patients who suffered injuries to the orbitofrontal cortex (Best et al., 2002; Ernst et al., 2003; Fairchild et al., 2009).

Bully-victims present similarities and differences with regard to both groups of victims and bullies. In the IGT, bully-victims were similar to controls and victims, but choose more advantage cards as compared to bullies. In SQD, bully-victims presented scores of prosocial behavior more similar to bullies than victims. A possible explanation is that the decreasing of prosocial behavior in bully-victims may be related to the dissemination of the aggression suffered. For Bandeira and Hutz (2010), bully-victims children are more likely to present depressive symptoms, anxiety, and externalizing behaviors, and unlike children in the bullies group, they are not popular but rather rejected by their peers.

The results of this study can be analyzed by considering the dichotomy proposed by Kerr and Zelazo (2004). These authors suggest the existence of hot (more closely related to motivation and emotional control) and cold (more closely related to logical-rational aspects of cognition) executive functions. If, on one hand, victims have experienced greater difficulties in tasks that require cold executive functions, then bullies have impaired “hot” executive functions.

This study includes certain limitations, such as the sample size and the fact that some children in the bully group have shown moderate levels of victimization. Another limitation of the study is the use of a self-reported scale, which can generate interference in the division of the groups, although this problem was minimized by the application of the ICU scale in children and parents. It should also be considered that the methodology used was transversal and causal inferences about the investigated aspects cannot be made. We suggest that future studies be conducted with larger samples, longitudinal studies and different cultures.

In addition to sociocultural variables, this study shows that executive functioning, including decision-making, can also play a relevant role in bullying behavior. This type of study may lead to the development of customized intervention strategies according to the profile of students in each school because not all behavioral issues are due to the same cause and not every victim responds to aggression in the same manner (Viding et al., 2011).

## Author Contributions

Conceived and designed the experiments: WM, NT-A, and CM. Performed the experiments: WM. Analyzed the data: WM, NT-A, and CM. Contributed with materials and analysis tools: WM, NT-A, CM, and LM-D. Wrote the paper: WM, NT-A, CM, and LM-D.

## Conflict of Interest Statement

The authors declare that the research was conducted in the absence of any commercial or financial relationships that could be construed as a potential conflict of interest.
